# Progression of chronic kidney disease: an illness-death model approach

**DOI:** 10.1186/s12882-017-0604-8

**Published:** 2017-06-30

**Authors:** Phisitt Vejakama, Atiporn Ingsathit, Mark McEvoy, John Attia, Ammarin Thakkinstian

**Affiliations:** 1 Section for Clinical Epidemiology and Biostatistics, Faculty of Medicine, Ramathibodi Hospital, Mahidol University, Bangkok, Thailand; 2 Division of Nephrology, Department of Medicine, Sunpasitthiprasong Hospital, Ubon Ratchathani, Thailand; 3 Centre for Clinical Epidemiology and Biostatistics, School of Medicine and Public Health, Faculty of Health and Medicine, University of Newcastle, Callaghan, NSW Australia

**Keywords:** Chronic kidney disease progression, CKD progression, Illness death model

## Abstract

**Background:**

Chronic kidney disease (CKD) is a major contributor to mortality in the general population. Understanding the factors that drive this process will help delay progression of CKD. The study aimed to estimate the risks of kidney failure and death prior to and after the development of kidney failure among patients with pre-existing CKD, and to identify potential prognostic factors.

**Method:**

Data were obtained from patients with CKD from Ubon Ratchathani province, Thailand from 1997 to 2011. The probability of each transition (i.e., CKD➔death (T1), CKD➔kidney failure (T2), and kidney failure➔death (T3)) was estimated using a competing risk model. A parametric survival model with restricted cubic spline function was applied to assess prognostic factors. Illness-death models were constructed for the 3 transitions. Among 32,106 patients with CKD, 5576 (17.4%), 4768 (14.9%), and 3056 (9.5%) respectively moved through T1, T2, and T3.

**Results:**

Diabetics had 22.6%, 13.5%, and 60.7% higher risks of T1, T2, and T3 than non-diabetics respectively (*p* < 0.001). Hypertension increased risks of T2 and T3 by 8.7% (*p* = 0.01) and 27.2% (*p* < 0.001), whereas cardiovascular disease increased risk of T1 and T3 by 76% and 42.7%, respectively (*p* = 0.01). Increasing HDL by 10 units respectively decreased risk of T1 and T2 by 0.5% (*p* = 0.002) and 1.4% (*p* < 0.001). In addition, renin-angiotensin blockade decreased risk of T2 by 35% (*p* < 0.001).

**Conclusions:**

Diabetes and cardiovascular disease are associated with increasing mortality among CKD patients both before and after the development of kidney failure while hypertension is associated with increasing mortality mainly following kidney failure. Diabetes and hypertension are associated with an elevated risk of kidney failure while elevated HDL levels and renin-angiotensin blockade appear protective.

**Electronic supplementary material:**

The online version of this article (doi:10.1186/s12882-017-0604-8) contains supplementary material, which is available to authorized users.

## Background

Chronic kidney disease (CKD) is one of the leading non-communicable diseases contributing to morbidity and mortality globally. In Thailand, the prevalence of CKD with estimated Glomerular Filtration Rate (eGFR) category 3 (G3) is about as common as diabetes, i.e., 8.6% [1] and 7.5% [2] respectively.

Prognostic factors for CKD progression have been studied [3]. Knowing these prognostic factors will potentially lead to identifying CKD risk properly and instituting treatments to delay CKD progression. Kidney failure and death are the common clinical endpoints of CKD progression; death from other causes is a competing risk in such analyses, but only 20 out of 132 studies (15.1%) appropriately accounted for death as a competing risk [3]. The Kaplan-Meier method might can lead to biased estimates of the cumulative incidence of kidney failure if the number of patients with the competing risk is high and this is not accounted for in the model [4]. A competing risk model handles this situation and might yield less bias in the estimated cumulative incidence function (CIF) than the Kaplan-Meier method [5–7]. However a competing risk model considers only the first occurrence of an event, e.g. transition from CKD to kidney failure or death, but not kidney failure to death. We therefore applied an illness-death model, which aimed to estimate the probabilities of three CKD transitions as follows: transition 1: G1-G4➔death; transition 2: G1-G4➔kidney failure; transition 3: kidney failure➔death. Prognostic factors for each transition were also assessed.

## Methods

### Participants

We used data from a retrospective cohort of patients with CKD living in 20 districts of Ubon Ratchathani province, Thailand. Computerized databases between 1997 and 2011 were retrieved, and death was then verified by linking these databases with the Thailand death registry. Subjects were eligible if they were 18 years or older, had been diagnosed with CKD, and had at least 1 year of follow-up. The flow of the cohort study and the data retrieval process have been published previously [8].

### Studied variables and measurements

Demographic data including age, gender, and body mass index (BMI) were measured at the time of diagnosis of CKD. Serum creatinine (Scr), which is standardized and calibrated every 3 months by the Department of Medical Science, Ministry of Public Health, was measured by each hospital laboratory unit. Results from the Modified Jaffe (MJ) method were converted to the Isotope Dilution Mass Spectrometry (IDMS) equivalent using the equation from the Thai SEEK study as described previously [1]. An eGFR was then calculated using the chronic kidney disease epidemiology collaboration (CKD-EPI) equation [9]. The urine analysis (UA) was done using a urine dipstick to test for proteinuria. The results were reported as negative or normal, trace (equivalent to micro-albuminuria) or 1+ or more (equivalent to macro-albuminuria). We used the Kidney Disease: Improving Global Outcomes (KDIGO) 2012 guideline to classify CKD [10]. GFR categories consisted of G1 (≥90 ml/min/1.73 m^2^), G2 (60–89 ml/min/1.73 m^2^), G3a (45–59 ml/min/1.73 m^2^), G3b (30–44 ml/min/1.73 m^2^), G4 (15–29 ml/min/1.73 m^2^), and G5 (<15 ml/min/1.73 m^2^).

Diabetes, hypertension, and cardiovascular disease (CVD) at the time of diagnosis of CKD were identified from the databases using ICD10 codes E10-E14 for diabetes, I10-I15 for hypertension, and I60-I69 for CVD. Patients were classified as users of renin angiotensin system (RAS) blockers if they had been prescribed angiotensin converting enzyme inhibitors (ACEIs) or angiotensin II receptor blockers (ARBs).

### All-cause mortality

Death certificates were retrieved from the Bureau of Strategy and Statistics, Ministry of Public Health database through to December 31, 2011. All deaths were registered and certified with no missing data.

### Statistical analysis

**Fig. 1:**
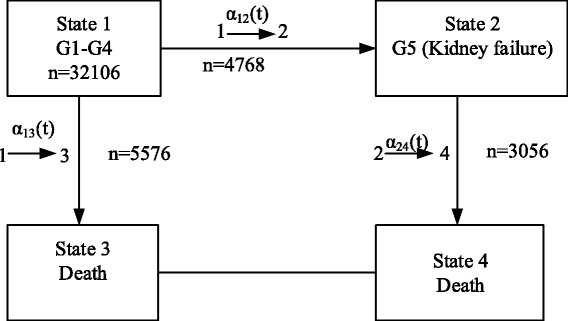
Illness-death model for CKD patients

A unidirectional illness-death model was created as described in Fig. 1. In this model, patients move from state i to state j over timeT_ij_; kidney failure was treated as an intermediate state and death was the absorbing state. Patients were initially entered into state 1 with a diagnosis of CKD G1-G4. They then moved to state 3 over time T_13_ (called transition 1), i.e., they died due to any cause other than kidney failure. Alternatively, some patients spent time T_12_ reaching state 2, i.e. diagnosis of kidney failure (called transition 2). After the diagnosis of kidney failure, patients were at risk of death, with time T_23_ (called transition 3), but some remained alive until the end of study.

A competing risk model with subdistribution hazard functions was used to estimate the CIF of transition 1 and transition 2 [5–7, 11], where kidney failure was the event of interest and all-cause mortality was a competing event. A Cox Proportional Hazards model was used to estimate probability of death after kidney failure for transition 3.

A flexible parametric survival model was applied to estimate the transition hazards, denoted as α_12_, α_13_, and α_23_ in Fig. 1 [12–14]. All three transition models were simultaneously fitted using restricted cubic spline functions to model the baseline hazard for each transition. Data were prepared in long format, in which data for each patient were expanded in 2 to 3 rows depending on the occurrence of kidney failure and death using the ‘illpred’ command in STATA [14]. Three transition-dummy variables (i.e., trans1 = 1 if transition =1, 0 otherwise; trans2 = 1 if transition = 2, 0 otherwise; trans3 = 1 if transition =3, 0 otherwise) were constructed and fitted into the cubic-spline model as time-varying covariates, stratifying by transition.

Prognostic factors for kidney failure and death including age, gender, BMI, diabetes, hypertension, CVD, lipid profiles (i.e., total cholesterol, triglyceride, HDL, and LDL), and RAS blockade were considered for inclusion in the parametric survival models. Data for BMI, triglyceride, LDL, and HDL were missing in 12.5%, 29.3%, 31.2%, and 33.7%, respectively of participants, so these were imputed using multivariate chain equations assuming data were missing at random [15, 16]. Linear regression models with 100 imputations were constructed to predict missing data and their averages were used for further analysis [17].

A univariate analysis was performed by adding each prognostic factor in the cubic spline regression. The main effect of each factor was fitted along with time-varying transitional variables (i.e., trans1, trans2, and trans3). A likelihood ratio test was applied to assess whether these main effects were significant or if the trend was significant. Variables whose *p* value was less than 0.10 for this step were simultaneously included in a multivariate model. In addition, we assessed whether these main effects varied across transitions; interactions between prognostic factors and transitional variables (i.e., trans1, trans2, and trans3) were fitted. Hazard ratios (HR) along with 95% confidence interval (CI) were then estimated by exponentiating coefficients. In addition, a Cox proportional Hazard model stratified by transition was also applied. All analyses for prognostic factors of CKD progression were performed using stpm2 and stpm2illd commands in STATA version 13.0. *P* values less than 0.05 were considered to be statistically significant.

## Results

Approximately 1.3 million people were screened for CKD between 1997 and 2011, and 32,106 were found to have the condition. The majority were females (63.7%); mean age and BMI were respectively 63.5 (SD = 12.8) years and 22.7 (SD = 4.3) kg/m^2^. Among all patients with CKDs, 46.8%, 42.9%, and 13.6% had diabetes, hypertension, and CVD, respectively (Table 1).

**Table 1:** Baseline characteristics of CKD patients

Characteristics	CKD *N* = 32,106
Follow-up time, years, median (range)	4.4 (0.3, 14.3)
Age, year, mean (SD)	63.5 (12.8)
Male, no (%)	11,707 (36.3)
eGFR, mean (SD)	46.9 (20.9)
Albuminuria category, no (%)	
A1	12,037 (49.3)
A2	6852 (28.1)
A3	5518 (22.6)
BMI, kg/m^2^, mean (SD)	22.7 (4.3)
Smoking, no (%)	1821 (8.5)
Co-morbidity, no (%)	
Hypertension	13,801 (42.9)
Diabetes	15,032 (46.8)
CVD	4371 (13.6)
Total cholesterol, mean (SD)	184.7 (53.7)
Triglyceride, median (range)	223.9 (122.6)
LDL, mean (SD)	115.2 (36.6)
HDL, mean (SD)	39.0 (10.1)
RAS blockade use^a^, no (%)	6949 (21.6)

^a^renin-angiotensin system

As described in Fig. 1, 32,106 subjects were classified as CKD stage G1 to G4 at enrollment and thus entered into state 1. These subjects were at risk for kidney failure (state 2) or for death without kidney failure (state 3); 4768 (14.9%) and 5576 (17.4%) moved through the former and the latter, respectively. For those 4768 subjects who reached state 2, 3056 (64.1%) died (state 4) whereas 1712 (35.9%) were still alive at the end of the study.

**Fig. 2:**
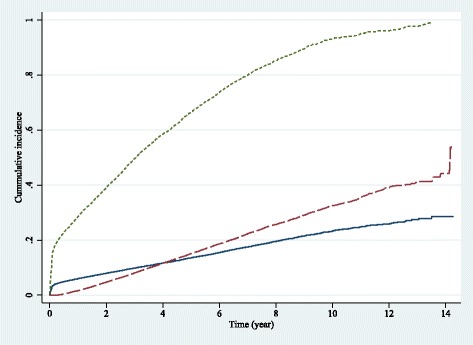
Cumulative incidence functions for 3 transitions. Transition 1: CKD➔Death . Transition 2: CKD➔Kidney failure . Transition 3: Kidney failure➔Death

A CIF for each transition was estimated and is reported in Fig. 2. The 2-, 5-, and 10-year probabilities of transition 1 were respectively 4.7%, 15.1%, and 32.5%. The 2-, 5-, and 10-year probabilities of transition 2 were 7.9%, 13.5%, and 23.3%, respectively. The corresponding probabilities of transition 3 were 39.0%, 66.4%, and 93.1%, respectively.

Each prognostic factor was fitted in a cubic spline regression assuming constant and varying effects on each transition. The two models were compared using a likelihood ratio test, indicating the model with varying effects was a better fit than that with constant effects (see Additional file 1: Table S1). The prognostic effects on each transition are described in Table 2. Every 10 year increase in age increased the risks of death before and after kidney failure by 5.1% (HR = 1.051: 95% CI: 1.048, 1.054) and 0.5% (HR = 1.005: 95% CI: 1.002, 1.008), respectively. However, age had no effect on kidney failure (HR = 0.999, 95% CI: 0.997, 1.002). Males were respectively 47.1% (HR = 1.471, 95% CI: 1.393, 1.553) and 29.7% (HR = 1.297, 95% CI: 1.201, 1.400) more likely to die before and after kidney failure when compared to females. In contrast, the risk of developing kidney failure was 7.8% (HR = 0.922, 95% CI: 0.866, 0.981) lower in males than females. BMI was significantly associated with all 3 transitions; for every one unit increase in BMI, the risks of death before kidney failure, kidney failure, and death after kidney failure decreased by 5.3% (HR = 0.947, 95% CI: 0.940, 0.954), 5.9% (HR = 0.941, 95% CI: 0.934, 0.948), and 2.3% (HR = 0.977, 95% CI: 0.968, 0.987), respectively.

**Table 2:** Prognostic factors of kidney failure and death through three transitions: Illness-death model

Transitions	Factors	Coefficients	SE	Z	P > |t|	HR	LL	UL
CKD➔Death	Age	0.0497	0.0013	37.31	<0.001	1.051	1.048	1.054
	Male/Female	0.3861	0.0277	13.938	<0.001	1.471	1.393	1.553
	BMI	-0.0542	0.0038	−14.308	<0.001	0.947	0.940	0.954
	HDL	-0.0052	0.0017	−3.033	0.002	0.995	0.991	0.998
	DM	0.2034	0.0302	6.736	<0.001	1.226	1.155	1.300
	HT	-0.0902	0.031	−2.909	0.004	0.914	0.860	0.971
	CVD	0.5651	0.0333	16.959	<0.001	1.760	1.648	1.878
	RAS	-0.0734	0.0393	−1.868	0.062	0.929	0.860	1.004
CKD➔Kidney failure	Age	−0.0007	0.0012	−0.601	0.548	0.999	0.997	1.002
	Male/Female	−0.0815	0.0317	−2.574	0.01	0.922	0.866	0.981
	BMI	-0.0608	0.004	−15.355	<0.001	0.941	0.934	0.948
	HDL	-0.0143	0.0019	−7.422	<0.001	0.986	0.982	0.990
	DM	0.1264	0.0318	3.979	<0.001	1.135	1.066	1.208
	HT	0.0836	0.0333	2.51	0.012	1.087	1.018	1.161
	CVD	-0.0998	0.0462	−2.16	0.031	0.905	0.827	0.991
	RAS	-0.4301	0.0449	−9.582	<0.001	0.650	0.596	0.710
Kidney failure➔death	Age	0.0051	0.0017	3.036	0.002	1.005	1.002	1.008
	Male/Female	0.2598	0.0391	6.651	<0.001	1.297	1.201	1.400
	BMI	-0.023	0.0049	−4.647	<0.001	0.977	0.968	0.987
	HDL	0.001	0.0019	0.545	0.586	1.001	0.997	1.005
	DM	0.4741	0.0404	11.744	<0.001	1.607	1.484	1.739
	HT	0.2403	0.0416	5.769	<0.001	1.272	1.172	1.380
	CVD	0.3554	0.0558	6.373	<0.001	1.427	1.279	1.592
	RAS	0.0974	0.0551	1.769	0.077	1.102	0.990	1.228

Our models suggest that diabetic subjects are at higher risk of all 3 transitions when compared to non-diabetic subjects; the risks were 22.6% (HR = 1.226: 95% CI: 1.155, 1.300), 13.5% (HR = 1.135066 95% CI: 1.066, 1.208), and 60.7% (HR = 1.607: 95% CI: 1.484, 1.739) higher for death before kidney failure, kidney failure, and death after kidney failure, respectively. Comparing the risk of death in diabetes between transition 3 and transition 1 suggested that the risk of death was 31.1% (HR = 1.311, 95% CI: 1.187, 1.447) higher in diabetes with kidney failure than diabetes without kidney failure.

Hypertensive subjects had 8.7% (HR = 1.087, 95% CI: 1.018, 1.161) and 27.2% (HR = 1.272, 95% CI: 1.172, 1.380) higher risk of kidney failure and death after kidney failure, respectively. Conversely, the risk of death for hypertensive patients before kidney failure was about 8.6% lower (HR = 0.914, 95% CI: 0.860, 0.971).

CVD significantly increased risks of death either before or after kidney failure when compared to non-CVD subjects. The risks of the former and the latter were about 76.0% (HR = 1.760, 95% CI: 1.648, 1.878) and 42.7% (HR = 1.427, 95% CI: 1.279, 1.592) higher, respectively. The risk of kidney failure was 9.5% lower in CVD than non-CVD (HR = 0.905, 95% CI: 0.827, 0.991).

Higher HDL levels carried lower risks of kidney failure, and for every 10 unit increase in HDL level, the risks of kidney failure and death before kidney failure decreased by 13.3% (HR = 0.867, 95% CI: 0.834, 0.900) and 5.1% (HR = 0.949, 95% CI: 0.917, 0.982), but the risk of death after kidney failure was not significant (HR = 1.001, 95% CI: 0.997, 1.005).

RAS blockade decreased the risk of kidney failure significantly (HR = 0.650, 95% CI: 0.596, 0.710) and showed trend in reduce the risk of death before kidney failure (HR = 0.929, 95% CI: 0.860, 1.004), whereas it increased risk of death after kidney failure (HR = 1.102, 95% CI: 0.990, 1.228) but this was not significant.

A sensitivity analysis was performed using a Cox Proportional Hazard regression model separately for each transition. Results were very similar to the cubic spline regression model, except that CVD in transition 2 was not significant in the Cox model but it was significant in the cubic spline model (HR = 0.926; 95% CI: 0.845, 1.013 vs 0.905; 95% CI 0.827, 0.991, see Additional file 1: Table S2).

In addition, a sensitivity analysis was performed by excluding CKD category G1/G2 (about 13.3% of patients) from the parametric survival model because these patients can revert to having normal eGFR over time. Results were very similar, except for transition 2 (CKD➔Kidney failure), in which age, sex, diabetes, and hypertension became non-significant (see Additional file 1: Table S3).

## Discussion

This study was conducted to assess the progression of CKD to kidney failure and/or death using the illness-death model approach. The model suggested that the 2-, 5-, and 10-year probabilities of kidney failure were 7.9%, 13.5%, and 23.3%, respectively. The risks of death increased sharply after kidney failure compared to death before kidney failure with the corresponding probabilities of 39.0%, 66.4%, and 93.1% versus 4.7%, 15.1%, and 32.5%, respectively.

Age, gender, BMI, diabetes, hypertension, CVD, HDL, and RAS blockade were prognostic factors in all three transitions. For every 10 year increase in age, the risk of death before and after kidney failure increased approximately 64% and 5%, respectively. This implies that age is a greater determinant of mortality before CKD than after. Not surprisingly, age was not associated with kidney failure, likely due to the fact that age was already taken into account when estimating eGFR. Males were more likely to die both before and after kidney failure, but they tended to develop kidney failure less than females. Higher BMI significantly decreased the risks of CKD progression in all respects. This paradoxical inverse association between BMI and mortality was consistent with findings obtained from large-scale studies [18, 19]; this may be explained by the fact that BMI is a marker of better health generally or that underweight is a marker of end-stage disease. Higher HDL decreased the risks of kidney failure and death before kidney failure, but had no effect on death after kidney failure.

Diabetes increased the risks of CKD progression in all respects. The effect of diabetes on death was more pronounced after developing kidney failure, i.e., approximately one third higher after kidney failure than before kidney failure. Hypertension was associated with increased risks of kidney failure and death after kidney failure, i.e., CKD progression was greater in hypertensive than non-hypertensive patients, but was associated with lower risk of death before kidney failure. A possible explanation for this inverse epidemiology might be survival bias; patients with more severe hypertension progressed more rapidly to kidney failure, leaving those with better health and more favorable prognosis at the pre-kidney failure stage to die of other causes. Alternatively, hypertensive patients without kidney failure also tend to be screened more for risks of CVD, including more frequent lipid testing and EKG monitoring, and thus have a higher chance of receiving effective medications for CVD prevention, including statins (83% vs 27%), and anti-platelet agents (58% vs 24%).

CVD was a significant predictor of death either before or after kidney failure, but was associated with lower risk of kidney failure. Our results also indicated a reno-protective effect of RAS blockade, i.e., a substantially lower risk of kidney failure by approximately 35%. However, its effect on death was very small and of borderline significance, i.e., lower risk for death before kidney failure but higher risk after kidney failure when compared to no RAS blockade. Effect of RAS blockade on death after kidney failure might be due to interaction with other co-morbidities (i.e., people with diabetes, CVD, dyslipidemia tend to be prescribed these agents more), but unfortunately, these interaction effects could not be detected (data not shown). Our results could still be affected by residual confounding and using other methods such as a counterfactual framework approach, inverse probability weighting, augmented inverse probability weighing, or propensity score matching would be useful [20].

Our findings suggest a rate of kidney failure of approximately 38/1000 person-years, which is lower than previous findings (83/1000 person-years [21]) and lower than renal replacement therapy (RRT) rates (139/1000 person-years [22]). Unlike these studies which estimated progression of more advanced CKD, our study assessed the full course of CKD progression starting from the G1 stage to kidney failure.

Although our estimate of death before kidney failure was similar to previous findings, i.e. 42 vs 59 [21] vs 39/1000 person-years [22], our death after kidney failure was nearly 10 times higher (304 vs 32/1000 person-years) [22]. This was probably due to the fact that our patients had less access to RRT after the diagnosis of kidney failure. In fact, the universal health coverage scheme for Thailand has provided RRT under a peritoneal dialysis first-policy only since 2008. Prior to this period, the access rate to RRT was less than 1%, and only about 0.05% of patients received kidney transplants. The RRT rate has increased sharply since 2008 to 28% in 2011. As a result, the risk of death was about 55% lower in those who received RRT than those who did not.

Among all the factors influencing progression from CKD to kidney failure, RAS blockade seems to be the most promising for clinical practice. The reno-protective effects of RAS blockade have been well demonstrated by a recent systematic review and meta-analysis of randomized controlled trials [23]. Unfortunately, only one fifth of our CKD subjects received RAS blockers and the majority of these received them at the G3 stage or higher.

Some of our findings were unexpected. For instance, we found protective effects of BMI (for all transitions), hypertension (for death before kidney failure), and CVD (for kidney failure), but adverse effects of RAS (on death after kidney failure). These findings may be due to methodological issues: effect modification and multi-collinearity. Effect modification of these factors were therefore explored (data not shown). Results suggested that higher BMI was associated with a higher risk of kidney failure but not of death, before and after kidney failure in diabetes patients. Hypertension risk was also modified by diabetes; the risk for kidney failure and death before kidney failure increased 73% and 31%, respectively. In addition, hypertension was also modified by CVD; there was an increased risk of kidney failure of 15% but not for death before and after kidney failure. Furthermore, these factors may be mediators themselves or may be mediated by other factors, so considering them in the same equation may cause inconsistency in the direction of association [24]. Causal effects of these factors should be determined using a mediation analysis to decompose direct and indirect effects of these factors on death [24, 25]. These variables were also highly correlated and multi-collinearity might result in invalid estimations, particularly for the variance of coefficients. This was explored by including each variable one by one in the model and seeing how the variance of each variable already contained in the model changed (see Additional file 1: Table S4). Results showed very small changes in variances and therefore multi-collinearity should be minimal.

It is common in research on chronic diseases to have more than one outcome of interest. In our example CKD progression can lead not only to kidney failure, but also to death before or after kidney failure. We therefore applied illness-death transition models, using parametric survival regression to model the baseline hazard [11, 12, 14, 26]. The model allowed us to determine the risk of occurrence of the three events simultaneously by fitting 3 transitions as time-varying variables in the model. A conventional Cox Proportional Hazard regression model was also applied for each transition separately, which yielded similar results to the parametric survival model.

Our study has some strengths. We have provided a picture of CKD progression using a large dataset with a median follow up time of 4.4 years. An annual CKD screen consisting of urine protein dip plus serum creatinine test, should be generalizable in other settings. The large dataset with long-term follow up allowed us to estimate the 2-, 5-, and 10-year probabilities of all 3 transitions. The use of appropriate statistical methods taking into account competing risks and interactions between prognostic factors and transitions should result in valid results. However, our study also had some limitations. This study was a retrospective cohort in which the data were retrieved from databases capturing routine practice. Data quality was not as good as for a dedicated prospective cohort and some variables contained missing data. Information on treatments of co-morbidity and treatments of CKD itself were lacking. This may result in biased prognostic effects of the studied co-variables.

## Conclusions

Our study has identified prognostic factors affecting three major transitions of CKD progression. Risk of death was dramatically increased after developing kidney failure. Diabetes and CVD increased risks of death before and after kidney failure. Hypertension increased the risk of death after kidney failure. Diabetes and hypertension increased the risk of kidney failure whereas renin-angiotensin blockade and HDL decreased the risk.

## Additional file

Additional file 1: Table S1. Likelihood ratio test of the models assuming constant versus varied effects of covariable on each of three transitions. **Table S2.** Prognostic factors of kidney failure and death through three transitions: Illness-death model by Cox Proportional Hazard regression analysis. **Table S3.** Prognostic factors of kidney failure and death through three transitions for CKD patients without G1-G2: Illness-death model. **Table S4.** Assess multi-colinearity for each transition. (DOCX 39 kb)

## Abbreviations

ACEIs: Angiotensin converting enzyme inhibitors
ARB: Angiotensin II receptor blocker
BMI: Body mass index
CI: Confidence interval
CIF: Cumulative incidence function
CKD: Chronic kidney disease
CKD-EPI: Chronic kidney disease epidemiology collaboration
CVD: Cardio-vascular disease
eGFR: Estimated glomerular filtration rate
HR: Hzard ratios
IDMS: Isotope dilution mass spectrometry
KDIGO: Kidney disease: improving global outcomes
MJ: Modified Jaffe
RAS: Renin angiotensin system
RRT: Renal replacement therapy
Scr: Serum creatinine
UA: Urine analysis

## References

[1] Ingsathit A, Thakkinstian A, Chaiprasert A, Sangthawan P, Gojaseni P, Kiattisunthorn K (2010). Prevalence and risk factors of chronic kidney disease in the Thai adult population: Thai SEEK study. Nephrol Dial Transplant.

[2] Aekplakorn W, Chariyalertsak S, Kessomboon P, Sangthong R, Inthawong R, Putwatana P, et al., Thai National Health Examination Survey IVSG. Prevalence and management of diabetes and metabolic risk factors in Thai adults: the Thai National Health Examination Survey IV, 2009. Diabetes Care. 2011;34(9):1980–5. 10.2337/dc11-0099 PMC3161276 21816976

[3] Boucquemont J, Heinze G, Jager KJ, Oberbauer R, Leffondre K (2014). Regression methods for investigating risk factors of chronic kidney disease outcomes: the state of the art. BMC Nephrol.

[4] Jager KJ, Stel VS, Zoccali C, Wanner C, Dekker FW (2010). The issue of studying the effect of interventions in renal replacement therapy -- to what extent may we be deceived by selection and competing risk?. Nephrol Dial Transplant.

[5] Pintilie M (2007). Analysing and interpreting competing risk data. Stat Med.

[6] Lau B, Cole SR, Gange SJ (2009). Competing risk regression models for epidemiologic data. Am J Epidemiol.

[7] Andersen PK, Geskus RB, de Witte T, Putter H (2012). Competing risks in epidemiology: possibilities and pitfalls. Int J Epidemiol.

[8] Vejakama P, Ingsathit A, Attia J, Thakkinstian A (2015). Epidemiological study of chronic kidney disease progression: a large-scale population-based cohort study. Medicine (Baltimore).

[9] Levey AS, Stevens LA, Schmid CH, Zhang YL, Castro AF, Feldman HI (2009). A new equation to estimate glomerular filtration rate. Ann Intern Med.

[10] National Kidney F (2012). KDOQI Clinical Practice Guideline for Diabetes and CKD: 2012 Update. Am J Kidney Dis.

[11] Putter H, Fiocco M, Geskus RB (2007). Tutorial in biostatistics: competing risks and multi-state models. Stat Med.

[12] Royston P, Parmar MK (2002). Flexible parametric proportional-hazards and proportional-odds models for censored survival data, with application to prognostic modelling and estimation of treatment effects. Stat Med.

[13] Nelson CP, Lambert PC, Squire IB, Jones DR (2007). Flexible parametric models for relative survival, with application in coronary heart disease. Stat Med.

[14] SR H (2013). Flexible parametric illness-death models. Stata J.

[15] Rubin DB, Schenker N (1991). Multiple imputation in health-care databases: an overview and some applications. Stat Med.

[16] White IR, Royston P, Wood AM (2011). Multiple imputation using chained equations: Issues and guidance for practice. Stat Med.

[17] van Buuren S, Boshuizen HC, Knook DL (1999). Multiple imputation of missing blood pressure covariates in survival analysis. Stat Med.

[18] Leavey SF, McCullough K, Hecking E, Goodkin D, Port FK, Young EW (2001). Body mass index and mortality in 'healthier' as compared with 'sicker' haemodialysis patients: results from the Dialysis Outcomes and Practice Patterns Study (DOPPS). Nephrol Dial Transplant.

[19] Johansen KL, Young B, Kaysen GA, Chertow GM (2004). Association of body size with outcomes among patients beginning dialysis. Am J Clin Nutr.

[20] G C (2014). treatrew: A user-written command for estimating average treatment effects by reweighting on the propensity score. Stata J.

[21] De Nicola L, Chiodini P, Zoccali C, Borrelli S, Cianciaruso B, Di Iorio B (2011). Prognosis of CKD patients receiving outpatient nephrology care in Italy. Clin J Am Soc Nephrol.

[22] Barbour SJ, Er L, Djurdjev O, Karim M, Levin A (2010). Differences in progression of CKD and mortality amongst Caucasian, Oriental Asian and South Asian CKD patients. Nephrol Dial Transplant.

[23] Vejakama P, Thakkinstian A, Lertrattananon D, Ingsathit A, Ngarmukos C, Attia J (2012). Reno-protective effects of renin-angiotensin system blockade in type 2 diabetic patients: a systematic review and network meta-analysis. Diabetologia.

[24] Lange T, Hansen JV (2011). Direct and indirect effects in a survival context. Epidemiology.

[25] VanderWeele TJ (2011). Causal mediation analysis with survival data. Epidemiology.

[26] PC L (2009). Further development of felxible parametric models for survival analysis. Stata J.

