# Supplementary material for: Progression of chronic kidney disease: an illness-death model approach
Source: BMC Nephrol. 2017 Jun 30;18:205. doi: 10.1186/s12882-017-0604-8 (PMC5493086; doi:10.1186/s12882-017-0604-8)
Supplement: Additional file 1: Table S1. — Likelihood ratio test of the models assuming constant versus varied effects of covariable on each of three transitions. Table S2. Prognostic factors of kidney failure and death through three transitions: Illness-death model by Cox Proportional Hazard regression analysis. Table S3. Prognostic factors of kidney failure and death through three transitions for CKD patients without G1-G2: Illness-death model. Table S4. Assess multi-colinearity for each transition. (DOCX 39 kb) [file 12882_2017_604_MOESM1_ESM.docx]

**Table S1. Likelihood ratio test of the models assuming constant versus varied effects of**

**covariable on each of three transitions**

| Factors | LL of constant effect models | LL of interaction models | LR test | Degree of freedom | P value |
| --- | --- | --- | --- | --- | --- |
| Age | -40291.3 | -39676.35 | 1229.91 | 12-10 | <0.001 |
| Male/Female | -40837.87 | -40731.98 | 211.79 | 12-10 | <0.001 |
| BMI | -40328.34 | -40118.76 | 419.16 | 12-10 | <0.001 |
| HDL | -40899.8 | -40864.91 | 69.78 | 12-10 | <0.001 |
| DM | -40924.57 | -40799.61 | 249.92 | 12-10 | <0.001 |
| HT | -40922.94 | -40865.91 | 114.06 | 12-10 | <0.001 |
| CVD | -40776.92 | -40647.08 | 259.68 | 12-10 | <0.001 |
| RAS | -40897.1 | -40825.46 | 143.28 | 12-10 | <0.001 |

LL, log-likelihood; LR, likelihood ratio

**Table S2. Prognostic factors of kidney failure and death through three transitions:**

**Illness-death model by Cox Proportional Hazard regression analysis**

| Factors | Coefficient | SE | Z | P | HR | LL | UL |
| --- | --- | --- | --- | --- | --- | --- | --- |

| Transition 1: CKD🡪Death |
| --- |

| Age | 0.0502 | 0.0013 | 37.608 | <0.001 | 1.051 | 1.049 | 1.054 |
| --- | --- | --- | --- | --- | --- | --- | --- |
| Male/Female | 0.3844 | 0.0277 | 13.868 | <0.001 | 1.469 | 1.391 | 1.551 |
| BMI | -0.0541 | 0.0038 | -14.281 | <0.001 | 0.947 | 0.940 | 0.954 |
| HDL | -0.0052 | 0.0017 | -3.008 | 0.003 | 0.995 | 0.991 | 0.998 |
| DM | 0.2071 | 0.0302 | 6.857 | <0.001 | 1.230 | 1.159 | 1.305 |
| HT | -0.0833 | 0.031 | -2.684 | 0.007 | 0.920 | 0.866 | 0.978 |
| CVD | 0.5705 | 0.0333 | 17.11 | <0.001 | 1.769 | 1.657 | 1.889 |
| RAS | -0.0641 | 0.0394 | -1.627 | 0.104 | 0.938 | 0.868 | 1.013 |

| Transition 2: CKD🡪Kidney failure |
| --- |

| Age | 0.001 | 0.0012 | 0.803 | 0.422 | 1.001 | 0.999 | 1.003 |
| --- | --- | --- | --- | --- | --- | --- | --- |
| Male/Female | -0.0741 | 0.0316 | -2.347 | 0.019 | 0.929 | 0.873 | 0.988 |
| BMI | -0.0628 | 0.0038 | -16.324 | <0.001 | 0.939 | 0.932 | 0.946 |
| HDL | -0.0152 | 0.0016 | -9.785 | <0.001 | 0.985 | 0.982 | 0.988 |
| DM | 0.1263 | 0.0316 | 3.991 | <0.001 | 1.135 | 1.066 | 1.207 |
| HT | 0.1084 | 0.0334 | 3.247 | 0.001 | 1.114 | 1.044 | 1.190 |
| CVD | -0.0766 | 0.0462 | -1.66 | 0.097 | 0.926 | 0.846 | 1.014 |
| RAS | -0.4018 | 0.045 | -8.937 | <0.001 | 0.669 | 0.613 | 0.731 |

| Transition 3: Kidney failure🡪Death |
| --- |

| Age | 0.0054 | 0.0017 | 3.214 | 0.001 | 1.005 | 1.002 | 1.009 |
| --- | --- | --- | --- | --- | --- | --- | --- |
| Male/Female | 0.2581 | 0.0391 | 6.604 | <0.001 | 1.294 | 1.199 | 1.398 |
| BMI | -0.0234 | 0.0049 | -4.727 | <0.001 | 0.977 | 0.967 | 0.986 |
| HDL | 0.001 | 0.0019 | 0.531 | 0.595 | 1.001 | 0.997 | 1.005 |
| DM | 0.4736 | 0.0404 | 11.72 | <0.001 | 1.606 | 1.483 | 1.738 |
| HT | 0.2481 | 0.0418 | 5.941 | <0.001 | 1.282 | 1.181 | 1.391 |
| CVD | 0.3579 | 0.0558 | 6.41 | <0.001 | 1.430 | 1.282 | 1.596 |
| RAS | 0.1052 | 0.0551 | 1.908 | 0.056 | 1.111 | 0.997 | 1.238 |

**Table S3. Prognostic factors of kidney failure and death through three transitions for CKD patients without G1-G2: Illness-death model**

| Transition | Factors | Coefficient | SE | Z | P>\|t\| | HR | LL | UL |
| --- | --- | --- | --- | --- | --- | --- | --- | --- |
| CKD🡪Death | Age | 0.0492 | 0.0015 | 32.223 | <0.001 | 1.050 | 1.047 | 1.054 |
|  | Male/Female | 0.3868 | 0.0292 | 13.241 | <0.001 | 1.472 | 1.390 | 1.559 |
|  | BMI | -0.0506 | 0.004 | -12.74 | <0.001 | 0.951 | 0.943 | 0.958 |
|  | HDL | -0.0053 | 0.0018 | -2.975 | 0.003 | 0.995 | 0.991 | 0.998 |
|  | DM | 0.1896 | 0.0319 | 5.937 | <0.001 | 1.209 | 1.135 | 1.287 |
|  | HT | -0.0937 | 0.0322 | -2.912 | 0.004 | 0.911 | 0.855 | 0.970 |
|  | CVD | 0.5512 | 0.0349 | 15.795 | <0.001 | 1.735 | 1.621 | 1.858 |
|  | RAS | -0.0717 | 0.0407 | -1.759 | 0.079 | 0.931 | 0.859 | 1.008 |
| CKD🡪Kidney failure | Age* | -0.0153 | 0.0013 | -11.339 | <0.001 | 0.985 | 0.982 | 0.987 |
|  | Male/Female* | -0.0127 | 0.0324 | -0.392 | 0.695 | 0.987 | 0.927 | 1.052 |
|  | BMI | -0.0609 | 0.0041 | -15.032 | <0.001 | 0.941 | 0.934 | 0.948 |
|  | HDL | -0.0143 | 0.0019 | -7.351 | <0.001 | 0.986 | 0.982 | 0.990 |
|  | DM* | -0.045 | 0.0327 | -1.375 | 0.169 | 0.956 | 0.897 | 1.019 |
|  | HT* | 0.0004 | 0.0337 | 0.011 | 0.991 | 1.000 | 0.936 | 1.069 |
|  | CVD | -0.1074 | 0.0471 | -2.28 | 0.023 | 0.898 | 0.819 | 0.985 |
|  | RAS | -0.4644 | 0.0462 | -10.051 | <0.001 | 0.629 | 0.574 | 0.688 |
| Kidney Failure🡪Death | Age | 0.0067 | 0.0017 | 3.907 | <0.001 | 1.007 | 1.003 | 1.010 |
|  | Male/Female | 0.2531 | 0.0399 | 6.346 | <0.001 | 1.288 | 1.191 | 1.393 |
|  | BMI | -0.0224 | 0.005 | -4.429 | <0.001 | 0.978 | 0.968 | 0.988 |
|  | HDL | 0.0014 | 0.0019 | 0.741 | 0.459 | 1.001 | 0.998 | 1.005 |
|  | DM | 0.4547 | 0.0412 | 11.034 | <0.001 | 1.576 | 1.453 | 1.708 |
|  | HT | 0.3432 | 0.0569 | 6.03 | <0.001 | 1.409 | 1.261 | 1.576 |
|  | CVD* | 0.0853 | 0.0567 | 1.506 | 0.132 | 1.089 | 0.975 | 1.217 |
|  | RAS* | 0.2561 | 0.0425 | 6.032 | <0.001 | 1.292 | 1.189 | 1.404 |

*Variables are different compared to the overall CKD analysis

**Table S4. Assess multi-colinearity for each transition**

| Model | | Factors | | | | Coefficient | | | SE | | | [95% Conf. | | | Interval] | | |
| --- | --- | --- | --- | --- | --- | --- | --- | --- | --- | --- | --- | --- | --- | --- | --- | --- | --- |
| Transition 1: CKD🡪Death | | | | | |  | | |  | | |  | | |  | | |
| Age | | Age | | | | 0.0577 | | | 0.0001 | | | 0.0575 | | | 0.0579 | | |
|  | | Age | | | | 0.0557 | | | 0.0001 | | | 0.0555 | | | 0.0560 | | |
| Add sex | | Male/Female | | | | 0.3682 | | | 0.0027 | | | 0.3629 | | | 0.3735 | | |
|  | | Age | | | | 0.0497 | | | 0.0001 | | | 0.0494 | | | 0.0499 | | |
| Add BMI | | Male/Female | | | | 0.3767 | | | 0.0027 | | | 0.3714 | | | 0.3820 | | |
|  | | BMI | | | | -0.0524 | | | 0.0003 | | | -0.0530 | | | -0.0517 | | |
|  | | Age | | | | 0.0495 | | | 0.0001 | | | 0.0493 | | | 0.0498 | | |
| Add HDL | | Male/Female | | | | 0.3720 | | | 0.0027 | | | 0.3667 | | | 0.3773 | | |
|  | | BMI | | | | -0.0533 | | | 0.0003 | | | -0.0539 | | | -0.0526 | | |
|  | | HDL | | | | -0.0061 | | | 0.0001 | | | -0.0063 | | | -0.0059 | | |
| Add DM | | Age | | | | 0.0509 | | | 0.0001 | | | 0.0506 | | | 0.0512 | | |
|  | | Male/Female | | | | 0.4018 | | | 0.0028 | | | 0.3964 | | | 0.4072 | | |
|  | | BMI | | | | -0.0575 | | | 0.0004 | | | -0.0582 | | | -0.0568 | | |
|  | | HDL | | | | -0.0054 | | | 0.0001 | | | -0.0056 | | | -0.0052 | | |
|  | | DM | | | | 0.1680 | | | 0.0029 | | | 0.1622 | | | 0.1738 | | |
| Add HT | | Age | | | | 0.0513 | | | 0.0001 | | | 0.0511 | | | 0.0516 | | |
|  | | Male/Female | | | | 0.4016 | | | 0.0028 | | | 0.3962 | | | 0.4070 | | |
|  | | BMI | | | | -0.0558 | | | 0.0004 | | | -0.0565 | | | -0.0551 | | |
|  | | HDL | | | | -0.0053 | | | 0.0001 | | | -0.0055 | | | -0.0051 | | |
|  | | DM | | | | 0.1730 | | | 0.0030 | | | 0.1672 | | | 0.1788 | | |
|  | | HT | | | | -0.0689 | | | 0.0028 | | | -0.0745 | | | -0.0634 | | |
| Add CVD | | Age | | | | 0.0499 | | | 0.0001 | | | 0.0496 | | | 0.0501 | | |
|  | | Male/Female | | | | 0.3845 | | | 0.0028 | | | 0.3791 | | | 0.3899 | | |
|  | | BMI | | | | -0.0545 | | | 0.0004 | | | -0.0552 | | | -0.0538 | | |
|  | | HDL | | | | -0.0053 | | | 0.0001 | | | -0.0055 | | | -0.0050 | | |
|  | | DM | | | | 0.1962 | | | 0.0030 | | | 0.1904 | | | 0.2020 | | |
|  | | HT | | | | -0.1131 | | | 0.0028 | | | -0.1187 | | | -0.1076 | | |
|  | | CVD | | | | 0.5615 | | | 0.0033 | | | 0.5550 | | | 0.5680 | | |
| Add RAS | | Age | | | | 0.0498 | | | 0.0001 | | | 0.0495 | | | 0.0500 | | |
|  | | Male/Female | | | | 0.3866 | | | 0.0028 | | | 0.3812 | | | 0.3920 | | |
|  | | BMI | | | | -0.0542 | | | 0.0004 | | | -0.0549 | | | -0.0535 | | |
|  | | HDL | | | | -0.0052 | | | 0.0001 | | | -0.0055 | | | -0.0050 | | |
|  | | DM | | | | 0.2030 | | | 0.0030 | | | 0.1972 | | | 0.2089 | | |
|  | | HT | | | | -0.0904 | | | 0.0031 | | | -0.0964 | | | -0.0843 | | |
|  | | CVD | | | | 0.5652 | | | 0.0033 | | | 0.5587 | | | 0.5717 | | |
|  | | RAS | | | | -0.0733 | | | 0.0039 | | | -0.0810 | | | -0.0656 | | |
| Transition 2: CKD🡪Kidney failure | | | | | | |  | | |  | | |  | | |  |  |
| Age | Age | | | 0.0043 | | | 0.0001 | | | 0.0040 | | | 0.0045 | | |  |  |
|  | Age | | | 0.0046 | | | 0.0001 | | | 0.0043 | | | 0.0048 | | |  |  |
| Add sex | Male/Female | | | -0.0919 | | | 0.0031 | | | -0.0979 | | | -0.0859 | | |  |  |
|  | Age | | | -0.0007 | | | 0.0001 | | | -0.0009 | | | -0.0005 | | |  |  |
| Add BMI | Male/Female | | | 0.3767 | | | 0.0027 | | | 0.3714 | | | 0.3820 | | |  |  |
|  | BMI | | | -0.0596 | | | 0.0004 | | | -0.0603 | | | -0.0589 | | |  |  |
|  | Age | | | -0.0013 | | | 0.0001 | | | -0.0016 | | | -0.0011 | | |  |  |
| Add HDL | Male/Female | | | -0.1101 | | | 0.0031 | | | -0.1161 | | | -0.1041 | | |  |  |
|  | BMI | | | -0.0611 | | | 0.0004 | | | -0.0618 | | | -0.0604 | | |  |  |
|  | HDL | | | -0.0151 | | | 0.0001 | | | -0.0154 | | | -0.0149 | | |  |  |
| Add DM | Age | | | -0.0010 | | | 0.0001 | | | -0.0012 | | | -0.0007 | | |  |  |
|  | Male/Female | | | -0.0928 | | | 0.0031 | | | -0.0989 | | | -0.0866 | | |  |  |
|  | BMI | | | -0.0632 | | | 0.0004 | | | -0.0639 | | | -0.0625 | | |  |  |
|  | HDL | | | -0.0147 | | | 0.0001 | | | -0.0149 | | | -0.0144 | | |  |  |
|  | DM | | | 0.0833 | | | 0.0031 | | | 0.0772 | | | 0.0893 | | |  |  |
| Add HT | Age | | | -0.0005 | | | 0.0001 | | | -0.0008 | | | -0.0003 | | |  |  |
|  | Male/Female | | | -0.0929 | | | 0.0031 | | | -0.0990 | | | -0.0867 | | |  |  |
|  | BMI | | | -0.0620 | | | 0.0004 | | | -0.0627 | | | -0.0612 | | |  |  |
|  | HDL | | | -0.0146 | | | 0.0001 | | | -0.0149 | | | -0.0144 | | |  |  |
|  | DM | | | 0.0864 | | | 0.0031 | | | 0.0803 | | | 0.0925 | | |  |  |
|  | HT | | | -0.0521 | | | 0.0031 | | | -0.0582 | | | -0.0461 | | |  |  |
| Add CVD | Age | | | -0.0003 | | | 0.0001 | | | -0.0006 | | | -0.0001 | | |  |  |
|  | Male/Female | | | 0.3845 | | | 0.0028 | | | 0.3791 | | | 0.3899 | | |  |  |
|  | BMI | | | -0.0623 | | | 0.0004 | | | -0.0631 | | | -0.0616 | | |  |  |
|  | HDL | | | -0.0146 | | | 0.0001 | | | -0.0149 | | | -0.0144 | | |  |  |
|  | DM | | | 0.0830 | | | 0.0031 | | | 0.0769 | | | 0.0890 | | |  |  |
|  | HT | | | -0.0459 | | | 0.0031 | | | -0.0520 | | | -0.0399 | | |  |  |
|  | CVD | | | -0.1145 | | | 0.0046 | | | -0.1235 | | | -0.1055 | | |  |  |
| Add RAS | Age | | | -0.0007 | | | 0.0001 | | | -0.0010 | | | -0.0005 | | |  |  |
|  | Male/Female | | | -0.0808 | | | 0.0031 | | | -0.0869 | | | -0.0746 | | |  |  |
|  | BMI | | | -0.0608 | | | 0.0004 | | | -0.0616 | | | -0.0601 | | |  |  |
|  | HDL | | | -0.0143 | | | 0.0001 | | | -0.0146 | | | -0.0141 | | |  |  |
|  | DM | | | 0.1257 | | | 0.0031 | | | 0.1195 | | | 0.1318 | | |  |  |
|  | HT | | | 0.0829 | | | 0.0033 | | | 0.0764 | | | 0.0894 | | |  |  |
|  | CVD | | | -0.0995 | | | 0.0046 | | | -0.1085 | | | -0.0904 | | |  |  |
|  | RAS | | | -0.4297 | | | 0.0045 | | | -0.4385 | | | -0.4210 | | |  |  |
| Transition 3: Kidney failure🡪Death | | | | | | | |  | | |  | | |  | | |  |
| Age | | | Age | | 0.0046 | | | 0.0002 | | | 0.0043 | | | 0.0049 | | |  |
|  | | | Age | | 0.0047 | | | 0.0002 | | | 0.0044 | | | 0.0050 | | |  |
| Add sex | | | Male/Female | | 0.2122 | | | 0.0038 | | | 0.2047 | | | 0.2197 | | |  |
|  | | | Age | | 0.0048 | | | 0.0002 | | | 0.0045 | | | 0.0051 | | |  |
| Add BMI | | | Male/Female | | 0.2123 | | | 0.0038 | | | 0.2048 | | | 0.2198 | | |  |
|  | | | BMI | | 0.0006 | | | 0.0004 | | | -0.0002 | | | 0.0015 | | |  |
|  | | | Age | | 0.0048 | | | 0.0002 | | | 0.0045 | | | 0.0052 | | |  |
| Add HDL | | | Male/Female | | 0.2128 | | | 0.0038 | | | 0.2053 | | | 0.2202 | | |  |
|  | | | BMI | | 0.0007 | | | 0.0004 | | | -0.0002 | | | 0.0015 | | |  |
|  | | | HDL | | 0.0006 | | | 0.0002 | | | 0.0003 | | | 0.0009 | | |  |
| Add DM | | | Age | | -0.0010 | | | 0.0001 | | | -0.0012 | | | -0.0007 | | |  |
|  | | | Male/Female | | 0.2978 | | | 0.0039 | | | 0.2902 | | | 0.3054 | | |  |
|  | | | BMI | | -0.0180 | | | 0.0005 | | | -0.0189 | | | -0.0171 | | |  |
|  | | | HDL | | 0.0016 | | | 0.0001 | | | 0.0013 | | | 0.0019 | | |  |
|  | | | DM | | 0.5391 | | | 0.0039 | | | 0.5314 | | | 0.5468 | | |  |
| Add HT | | | Age | | 0.0057 | | | 0.0002 | | | 0.0054 | | | 0.0060 | | |  |
|  | | | Male/Female | | 0.2748 | | | 0.0039 | | | 0.2672 | | | 0.2824 | | |  |
|  | | | BMI | | -0.0236 | | | 0.0005 | | | -0.0245 | | | -0.0227 | | |  |
|  | | | HDL | | 0.0013 | | | 0.0001 | | | 0.0010 | | | 0.0016 | | |  |
|  | | | DM | | 0.4853 | | | 0.0040 | | | 0.4774 | | | 0.4931 | | |  |
|  | | | HT | | 0.2921 | | | 0.0039 | | | 0.2845 | | | 0.2998 | | |  |
| Add CVD | | | Age | | 0.0050 | | | 0.0002 | | | 0.0047 | | | 0.0053 | | |  |
|  | | | Male/Female | | 0.2617 | | | 0.0039 | | | 0.2540 | | | 0.2693 | | |  |
|  | | | BMI | | -0.0228 | | | 0.0005 | | | -0.0237 | | | -0.0218 | | |  |
|  | | | HDL | | 0.0010 | | | 0.0001 | | | 0.0007 | | | 0.0013 | | |  |
|  | | | DM | | 0.4830 | | | 0.0040 | | | 0.4752 | | | 0.4908 | | |  |
|  | | | HT | | 0.2638 | | | 0.0039 | | | 0.2561 | | | 0.2714 | | |  |
|  | | | CVD | | 0.3534 | | | 0.0056 | | | 0.3425 | | | 0.3643 | | |  |
| Add RAS | | | Age | | 0.0051 | | | 0.0002 | | | 0.0048 | | | 0.0054 | | |  |
|  | | | Male/Female | | 0.2600 | | | 0.0039 | | | 0.2524 | | | 0.2677 | | |  |
|  | | | BMI | | -0.0230 | | | 0.0005 | | | -0.0239 | | | -0.0221 | | |  |
|  | | | HDL | | 0.0010 | | | 0.0001 | | | 0.0007 | | | 0.0013 | | |  |
|  | | | DM | | 0.4743 | | | 0.0040 | | | 0.4665 | | | 0.4822 | | |  |
|  | | | HT | | 0.2402 | | | 0.0042 | | | 0.2321 | | | 0.2484 | | |  |
|  | | | CVD | | 0.3558 | | | 0.0056 | | | 0.3448 | | | 0.3667 | | |  |
|  | | | RAS | | 0.0978 | | | 0.0055 | | | 0.0871 | | | 0.1086 | | |  |
